# Tumor Inhibitory Effect of Long Non-coding RNA LOC100505817 on Gastric Cancer

**DOI:** 10.3389/pore.2021.581542

**Published:** 2021-05-26

**Authors:** Lei Zheng, Liying Kang, Yan Cheng, Junli Cao, Lijie Liu, Hongmei Xu, Liming Gao

**Affiliations:** ^1^ Department of Oncology, The First Hospital of Qinhuangdao, Qinhuangdao, China; ^2^ Department of Oncology, Wuqing People Hospital, Tianjin, China; ^3^ Disinfection Supply Room, The First Hospital of Qinhuangdao, Qinhuangdao, China

**Keywords:** long non-coding RNA LOC100505817, wnt/β-catenin signaling pathway, gastric cancer, epithelial-mesenchymal transition, proliferation, invasion, migration, apoptosis

## Abstract

Gastric cancer (GC) is one of the major malignancies worldwide. Emerging evidence has revealed the potential involvement of long noncoding RNA (lncRNA) in human genetic disorders and cancer, but the role of LOC100505817 remains unknown. Thus, in this study, we isolated tissues from GC patients to characterize the functional importance of LOC100505817 in GC tumorigenesis. We also proposed a hypothesis that the regulation of Wnt/β-catenin pathway by LOC100505817 was regulated by miR-20a-mediated WT1. After the collection of cancer tissues and adjacent tissues were obtained from GC patients, expression of LOC100505817, Wnt/β-catenin pathway- and EMT-related genes was quantified. Ectopic expression and knockdown experiments were applied in order to investigate the protective role of LOC100505817 in the progression of GC. Subsequently, cell viability, flow cytometry for apoptosis and cell cycle were detected via CCK-8, while migration and invasion were determined using scratch test and Transwell assay respectively. Then interactions among LOC100505817, miR-20a and WT1 were explored by dual luciferase reporter gene assay, RNA pull down assay and RNA binding protein immunoprecipitation (RIP) assay. The results found poor expression LOC100505817 was poorly expressed in GC cells and tissues. Overexpressed LOC100505817 resulted in the significant reduction of cell proliferation, migration and invasion as well as the expression of Wnt2b, β-catenin, CyclinD1, N-cadherin, Vimentin and snail, while increased cell apoptosis along with the expression of E-cadherin. Wnt/β-catenin pathway and EMT in GC cells were suppressed by LOC100505817 through miR-20a-inhibted WT1. In summary, our results provided evidence suggesting that LOC100505817 inhibits GC through LOC100505817-mediated inhibition of Wnt/β-catenin pathway, that leads to the overall restraining of GC cell proliferation, migration and invasion through miR-20a-reduced WT1.

## Introduction

Gastric cancer (GC), one of the most prevalent cancers, remains to be the second most common cause of cancer-related mortality worldwide [1]. China accounts for 43% of all GC patients worldwide and therefore has the largest GC patient population in the world [2]. Several risk factors have been identified for the occurrence of GC, some of which include genetic factors, *helicobacter pylori* infection, unhealthy diet and smoking [3]. The clinical features for GC include a high risk of occurrence, unspecific clinical symptoms, increased invasive and metastatic potential, all of which contribute to the poor prognosis seen in GC patients [4]. Conventional diagnostic methods, including early diagnosis and intervention with surgical resection, have contributed to GC mortality reduction; while these methods have been found to have the highest efficacy in the management of GC, the overall survival rate remains relatively low [5, 6]. Thus, it is urgent to thoroughly explore the molecular mechanism underlying GC.

Long non-coding RNAs (lncRNAs), longer than 200 nucleotides, are recognized as a large proportion of transcriptomes results in RNA transcripts [7]. The importance of lncRNAs in tumor development, through their regulatory role on biological processes, has been highlighted previously [8]. For example, the lncRNAs, HOX transcript antisense RNA (HOTAIR), metastasis associated lung adenocarcinoma transcript 1 (MALAT-1) and H19 have all been reported as functional contributors of carcinogenesis or cancer growth [9–11]. The connection between lncRNAs and GC has been thoroughly investigated [12]. In the cytosol, lncRNA SNHG5 suppresses GC development by trapping MTA2 [13]. In addition, 30% of GC cases have been observed to have activated Wnt/β-catenin signaling pathway, which promote gastric carcinogenesis [14]. Furthermore, previous data have established that ectopic expression of lncRNA-C-terminal domain 903 (CTD903) results in the suppression of the Wnt/β-catenin signaling pathway activation and inhibits cell invasion and migration in colorectal cancer [15], and overexpressed lncRNA HNF1A-antisense 1 (HNF1A-AS1) activates the Wnt/β-catenin signaling pathway, thus promoting osteosarcoma cell proliferation and metastasis [16], implying the close association between lncRNAs and malignant diseases through the modulation of the Wnt/β-catenin signaling pathway. Moreover, the loss or down-regulation of E-cadherin is considered as an important epithelial-mesenchymal transition (EMT) marker [16]. During EMT, cadherin switches from E-cadherin to N-cadherin, which is expressed in mesenchymal cells. Thus, downregulated E-cadherin expression and up-regulated N-cadherin expression are observed during EMT, both of which can serve as key EMT biomarkers. EMT-related proteins including Snail-1, ZEB-1, E-cadherin, vimentin and β-catenin [17]. Thus, we put forward a hypothesis that LOC100505817 has a crucial function in GC via the Wnt/β-catenin signaling pathway and conducted the present studye with the purpose of inspecting the effect of LOC100505817 on GC cell proliferation, migration, invasion and apoptosis as well as EMT by targeting the Wnt/β-catenin signaling pathway.

## Materials and Methods

### Ethics Statement

The study was conducted under the approval of the Ethics Committee of the First Hospital of Qinhuangdao and in accordance with the principles of *Declaration of Helsinki*. Written informed consents were obtained from enrolled patients, in agreement for the tissue samples to be kept for research.

### Analysis of Microarray Data

NCBI (https://www.ncbi.nlm.nih.gov/geo/) stands as a public platform with stored gene expression datasets, the original sequence and records. Data retrieval revealed the GC gene chip GSE13911. We downloaded data from the GEO chip, and GSE13911 was identified to include 31 normal samples and 38 tumor samples. Meanwhile, the annotation information of the sequencing platform of GSE13911 microarray data was obtained in the details of GSE13911 microarray data in Gene Expression Omnibus (GEO) database: GPL570 [HG-U133_Plus_2] Affymetrix Human Genome U133 Plus 2.0 Array. The probe ID was converted to genesymbol by using the annotation information, Then, with the normal sample serving as the control, an empirical Bayes method was conducted to screen out differentially expressed genes (DEGs) based on “limma” package of the Bioconductor using R language [18] (The screening criteria was |log2FC| > 0.5 and *p. Val* < 0.05). The DEGs were finally annotated by the “annotate” package. *p* < 0.05 was indicative of statistical significance.

### Study Subject

Cancer tissues and adjacent tissues (≥10 cm away from the cancer tissues) were collected from 92 patients diagnosed with GC [19] at the First Hospital of Qinhuangdao between September 2011 and September 2013. There were 48 males and 44 females, within the age of 38–78 years, with a median age of 60 ± 8 years. None of the enrolled patients received chemotherapy or radiotherapy prior to the operation. Among the patients, 38 cases had tumor size <5 cm and 54 cases had tumor size ≥5 cm; 55 cases had lymph node metastasis (LNM) and 37 cases had no LNM; 50 cases had highly or moderately differentiated tumor and 42 cases had poorly or undifferentiated tumor; 32 cases was at stage I–II and 60 cases was at stage III–IV according to LNM staging [20]. The inclusion criteria were as follows: 1) patients confirmed by pathology and/or cytology with recurrence and metastasis after regular treatment; 2) patients with the physical condition rated with a 0–2 score according to Eastern Cooperative Oncology Group (ECOG) [21], and with an expected survival time of ≥3 months; 3) patients without major organ dysfunction. The exclusion criteria included: 1) patients treated with preoperative chemotherapy, radiotherapy or other anti-tumor therapies; 2) patients had other malignant tumors in other organs; 3) patients had incomplete pathological data. Each tissue sample was frozen in liquid nitrogen.

### Reverse Transcription Quantitative Polymerase Chain Reaction (RT-qPCR) [22–24]

About 100 mg tissues frozen with liquid nitrogen were added with 1 ml Trizol, and the homogenate was prepared. Extraction of the total RNA was conducted based on the instructions of Trizol reagent. RNA sample of 10 μl was collected and diluted 20 times with ultrapure water without RNA enzyme. The optical density (OD) values at 260 and 280 nm were read in an ultraviolet (UV) spectrophotometer to measure the concentration and purity of RNA. After that, a PrimeScript RT reagent Kit (RR047A,Takara, Japan) was utilized for RNA reverse transcription into cDNA. Mix reagent (5 μl; 4368702, Tideradar Beijing Technology Co., Ltd., Beijing, China) was mixed with RNase Free H_2_O of 10 μl in an eppendorf (EP) tube, centrifuged, and then placed into the PCR instrument (9700, Beijing Dingguo Changsheng Biotechnology Co., Ltd., Beijing, China). Moreover, the generated cDNA was preserved at 20°C. Then, 1 μg cDNA was transferred into a 96 well PCR plate, followed by addition of 10 μl SYBR Green Master Mix (product No. rr091a, Takara company, Japan) and the primers in each well. The primers were synthesized by Shanghai Invitrogen Company (Shanghai, China) (primer sequences are listed in Table 1). RT-qPCR experiment was carried out on an ABI7300 quantitative PCR instrument (Applied Biosystems, Carlsbad, CA, United States). The samples were stained with SYBR Green fluorescent dye (TaKaRa Biotechnology Dalian Co., Ltd., Dalian, Liaoning, China). The Q reaction conditions were as follows: pre denaturation at 95°C for 10 min, denaturation at 95°C for 10 s, annealing at 60°C for 20 s, extending for 34 s at 72°C, for 40 cycles, and then the final extension was carried out at 72°C for 5 min. Relative quantitative value was evaluated using the 2^−△△Ct^ method, with glyceraldehyde-3-phosphate dehydrogenase (GAPDH) serving as the internal reference [25]. The experiment was conducted in triplicate. This method was also applicable for the detection of mRNA expression of related genes in cells.

**TABLE 1:** Primer sequences for RT-qPCR.

Gene	Sequence
LOC100505817	F: 5′-CCA​CTT​TAT​TTG​TCG​GTA​CAA​CGA​GTG-3′
	R: 5′-GGG​AGG​TTC​CCC​GAT​CCG​AGT​CAC​GG-3′
Wnt 2b	F: 5′-CCT​GTA​GCC​AGG​GTG​AAC​TG-3′
	R: 5′-CGG​GCA​TCC​TTA​AGC​CTC​TT-3′
N-cadherin	F: 5′-CAA​CTT​GCC​AGA​AAA​CTC​CAG​G-3′
	R: 5′-ATG​AAA​CCG​GGC​TAT​CTG​CTC-3′
Snail	F: 5′-AAT​CGG​AAG​CCT​AAC​TAC​AGC​G-3′
	R: 5′-GTC​CCA​GAT​GAG​CAT​TGG​CA-3′
β-catenin	F: 5′-TGG​CAA​CCA​AGA​AAG​CAA​G-3′
	R: 5′-CTG​AAC​AAG​AGT​CCC​AAG​GAG-3′
CyclinD1	F: 5′-AAC​TAC​CTG​GAC​CGC​TTC​CT-3′
	R: 5′-CCA​CTT​GAG​CTT​GTT​CAC​CA-3′
E-cadherin	F: 5′-CCC​ACC​ACG​TAC​AAG​GGT​C-3′
	R: 5′-CTG​GGG​TAT​TGG​GGG​CAT​C-3′
Vimentin	F: 5′-CGC​CAG​ATG​CGT​GAA​ATG​G-3′
	R: 5′-ACC​AGA​GGG​AGT​GAA​TCC​AGA-3′
MMP2	F: 5′-AGC​ATG​TCC​CTA​CCG​AGT​CT-3′
	R: 5′-AAA​CAG​ATG​GCA​AAC​ACG​GC-3′
ZO-1	F: 5′-TGC​TGA​GTC​CTT​TGG​TGA​TG-3′
	R: 5′-AAT​TTG​GAT​CTC​CGG​GAA​GAC-3′
α-SMA	F: 5′- GCC​ATT​CAT​GTC​AGA​GCT​ACA​CT-3′
	R: 5′- CCT​GTG​TTG​TGG​TTT​ACA​CTG​G-3′
FN	F: 5′-GTG​TTG​GGA​ATG​GTC​GTG​GGG​AAT​G-3′
	R: 5′-CCA​ATG​CCA​CGG​CCA​TAG​CAG​TAG​C-3′
GAPDH	F: 5′-GGT​GGT​CTC​CTC​TGA​CTT​CAA​CA-3′
	R: 5′-GTT​GCT​GTA​GCC​AAA​TTC​GTT​GT-3′

Notes: RT-qPCR, reverse transcription quantitative polymerase chain reaction; F, forward primers; R, reverse primers; GAPDH, glyceraldehyde-3-phosphate dehydrogenase; MMP2, matrix metalloproteinase 2; ZO-1, zonula occludens-1; α-SMA, α-smooth muscle actin; FN, fibronectin.

### Western Blot Analysis

The GC tissues and adjacent tissues were ground into uniform fine powder with liquid nitrogen, and further ground into homogenate on an ice bath with 1 ml radioimmunoprecipitation assay (RIPA) lysis buffer (P0013B, Beyotime Biotechnology Co., Ltd., Shanghai, China) containing phosphatase and protease inhibitors. Next, tissues received treatment with protein lysis buffer at 4°C for 30 min at an interval of 10 min and subjected to a 10 min-centrifugation at 12,000 rpm at 4°C. The protein concentration was measured in accordance with the instructions of a bicinchoninic acid (BCA) kit (BCA1-1KT, Sigma, St Louis, MO, United States). Subsequently, the sample concentration was adjusted with deionized water. With separation gel and concentration gel containing 10% sodium dodecyl sulfate (SDS) prepared, the samples and sample loading buffer were thoroughly mixed, and boiled at 100°C for 5 min. After an ice-bath and centrifugation, 20 μg proteins were transferred into each lane using a micro sampler for electrophoresis separation. Then the proteins on the gel were transferred into nitrocellulose membranes. After a 1-h sealing at room temperature, the membranes were probed with the addition of rabbit anti-human antibodies purchased from ProSpec-Tany (Rehovot, Israel): Vimentin (SR0746, 1 : 200), β-catenin (SR5436, 1 : 500), CyclinD1 (CRM-006, 1 : 200), E-cadherin (SR0617, 1 : 500), N-cadherin (SR0609, 1 : 500) and snail (10494-1-AP, 1 : 200), and antibodies purchased from Abcam (Cambridge, UK): matrix metalloproteinase 2 (MMP2, ab92536), zonula occludens-1 (ZO-1, ab96587), α-smooth muscle actin (α-SMA, ab108424), fibronectin (FN, ab6328) as well as horseradish peroxidase (HRP)-conjugated rabbit anti-human antibody to the internal reference GAPDH (1:1,000). The membranes were sealed in bag, and placed in a shaking bed at 4°C overnight. The following day, the membranes were washed three times with Tris-buffered saline Tween-20 (TBST; 5 min each time) at room temperature, after which incubation was carried out with HRP-labeled secondary antibody, goat anti-rabbit antibody (P0265, Beyotime Biotechnology Co., Ltd., Shanghai, China) diluted with TBST at a ratio of 1 : 5000 for 1 h. After three TBST washes in a shaking bed (5 min each time) at room temperature, the membranes were soaked in chemiluminescence (ECL) reaction liquid (Pierce, Rockford, IL, United States) for 1 min. The membranes were treated with X-ray exposure under dark conditions, then developed, and finally fixed to observe the results. ImageJ software was employed for quantification. The relative level of protein was regarded as the ratio of the gray value of target band to that of the internal reference band, with GAPDH serving as the internal reference. The experiment was repeated three times. This method was also suitable for detection of protein expression of related genes in cells.

### Cell Grouping and Transfection

Human GC cell lines BGC823, AGS, MKN-45, SGC-7901 and MKN-28 (Shanghai cell bank, Shanghai, China) were exposed to Roswell Park Memorial Institute (RPMI) 1640 medium (22400089, GIBCO BRL, Grand Island, NY, United States) supplemented with 10% fetal bovine serum (FBS) and 1% penicillin/streptomycin. Then cells were respectively seeded into six-well plates (1 × 10^5^ cells each well), incubated in a 5% CO_2_ incubator at 37°C under the condition of saturated humidity. Upon reaching 80–90% confluence, the cells were passaged. After the removal of the medium, cells were rinsed with phosphate buffer saline (PBS), incubated with 0*.*25% trypsin for 2∼5 min, resuspended in 5 ml RPMI 1640 medium containing 10% FBS, then passaged again.

GC SGC-7901 cells in logarithmic growth phase were transfected and assigned into blank group (SGC-7901 cells without any transfection), the small interfering (si)-negative control (NC) group (SGC-7901 cells transfected with NC sequence of si-LOC100505817), the si-LOC100505817 group (SGC-7901 cells transfected with si-LOC100505817, sequence: GGA​GGG​UGC​UUG​ACA​AUA​AUU), the pGMLV-vector group (SGC-7901 cells transfected with the pGMLV empty vector), the pGMLV-LOC100505817 group (SGC-7901 cells transfected with pGMLV-LOC100505817), the si-NC group (SGC-7901 cells transfected with NC sequence of si-β-catenin), the si-β-catenin group (SGC-7901 cells transfected with si-β-catenin, sequence: CTC​GGG​ATG​TTC​ACA​ACC​GAA), and the pGMLV-LOC100505817 + oe-β-catenin group (SGC-7901 cells transfected with pGMLV-LOC100505817 and β-catenin overexpression plasmid) [26]. The overexpression plasmid pGMLV-SV40T and the silencing plasmid pGPU6/Neo were respectively purchased from Genomeditech (GM-0210SV01, Shanghai, China) and Shanghai GenePharma Co. Ltd. (Shanghai, China). The cells were reversely transfected with siRNAs (Qiagen, Hilden, Germany; Dharmacon/Thermo Fisher Scientific, Schwerte, Germany) using RNAiMAX (Invitrogen) or HiPerFect (Qiagen) [26]. The day prior to the transfection, SGC-7901 cells at logarithmic growth phase was plated into 12-well plates at a density of 1 × 10^5^ cells/well. Cells in each well were added with 800 μl serum-free medium once the cell confluence reached 50∼70%. The mixture of pGMLV empty vector/pGmLV-LOC100505817 and lipo2000, or si-LOC100505817/LOC100505817-NC and lipo2000 (11668027, Thermo Fisher Scientific Inc., Waltham, MA, United States) was added into 12-well plates for a 6-h culture. At the 48th h after transfection, the cells were observed under a fluorescence microscope. The cells were harvested, and the RNA and protein were extracted for subsequent experiments.

### Cell Counting Kit-8 (CCK8) Assay

The GC cells after transfection (2 × 10^3^ cells/ml) were seeded into 96-well plates and added with 100 μl culture medium into each well. Optical density (OD) values at the 12, 24, 48, and 72 h were detected. Then cells in each well were supplemented with 10 µl CCK8 reagent (C0037, 1: 10, Beyotime Biotechnology Co., Ltd., Shanghai, China) for a 2-h culture at 37°C. The microplate reader (Multiskan FC, Thermo Fisher Scientific Inc., Waltham, MA, United States) was adopted for reading with the OD value at 450 nm. Three parallel wells were set with the average value obtained. Cell viability was detected and a cell growth curve was plotted with time as the abscissa and the OD value as the ordinate.

### Scratch Test

The cells after transfection were seeded into 6-well plates (5 × 10^5^ cells/well). A sterile gun head was gently across the axis of the well once the cell confluence reached 90%. After the floated cells were rinsed with PBS, the cells were added with serum-free medium was added for a 0.5∼1 h culture for recovery. The cells were photographed at the 0^th^ and 24th h of culture. Finally, Image-Pro Plus Analysis (Media Cybernetics, Silver Spring, MD, United States) was employed to measure the distance of cell migration, with the average value obtained.

### Transwell Assay

Dissolved and diluted (with serum-free medium at the ratio of 1 : 3) Matrigel matrix glue (356234, BD Biosciences, Franklin Lakes, NJ, United States) was added into the apical chamber of Transwell (50 μl each well), and incubation was carried out for 30 min. Cell suspension (1 × 10^5^ cells/ml) was added into the apical chamber (serum-free medium), and the medium containing 10% FBS was supplemented into the basolateral chamber. After culture for 48 h, the cells that failed to invade were removed with cotton swabs. The cells were rinsed three times with PBS, fixed with 4% formaldehyde solution and stained with crystal violet. The number of cells crossing was regarded as the index of invasive ability. The cells that reached at the reverse side of the chamber through matrix pores during 24 h were observed. A total of four visual fields were randomly selected under a microscope (×200). The cells invaded were calculated and photographed. The mean number of cells was calculated in each visual field.

### Flow Cytometry

At the 48th h after transfection, the cells were washed once with PBS, treated with 0.25% trypsin, mechanically dissociated into mixed suspension with cell detached. The mixed suspension was subjected to a 5-min centrifugation at 1,000 r/min. After being washed twice with PBS, the cells were fixed for 30 min with 70% pre-cooled ethanol. After another centrifugation, the cells rinsed with PBS again, stained with 1% Propidium iodide (PI; Baoman Biological Technology Co., Ltd., Shanghai, China) containing Ribonuclease (RNase) for 30 min, and rinsed two times with PBS. The sample volume was adjusted to 1 ml by PBS, and the BD-Aria flow cytometer (FACSCalibur, Beckman Kurt, Miami, FL, United States) was utilized for cell cycle detection. Three parallel wells were set in each group. The red fluorescent at the excitation wavelength of 488 nm was detected for cell cycle measurement.

At the 48th h after transfection, the cells were incubated with ethylene diamine tetraacetic acid (EDTA)-free trypsin, and collected in a flow tube, and underwent centrifugation. After being rinsed three times with cold PBS, the cells were centrifuged again. On the basis of the instructions of the Annexin-V-fluorescein isothiocyanate (FITC) apoptosis assay kit (Beyotime Biotechnology Co., Ltd., Shanghai, China), Annexin-V-FITC/PI staining solution was prepared with Annexin-V-FITC, PI and 2-[4-(2-Hydroxyethyl)-1-piperazinyl] ethanesulfonic acid (HEPES) at the ratio of 1:2:50. With 1 × 10^6^ cells re-suspended per 100 μl staining solution, the samples were gently shaken and mixed. After a 15 min-incubation at room temperature, the samples were mixed thoroughly with 1 ml HEPES buffer. Cell apoptosis at the excitation wavelength of 525 and 620 nm was detected through band pass.

### Statistical Analysis

All data were processed using SPSS 21.0 software (IBM Corp, Armonk, NY, United States). The measurement data was depicted by mean ± standard deviation. Comparisons between GC tissues and adjacent tissues were performed by *t* test, and multi-group comparisons were carried out by one-way analysis of variance (ANOVA). Comparisons among multiple groups at different groups were analyzed by two-way ANOVA, followed by Bonferroni post-hoc test. *p* < 0.05 was indicative of significant difference.

## Results

### Downregulated LOC100505817 Contributes to the Development of GC

**FIGURE 1:**
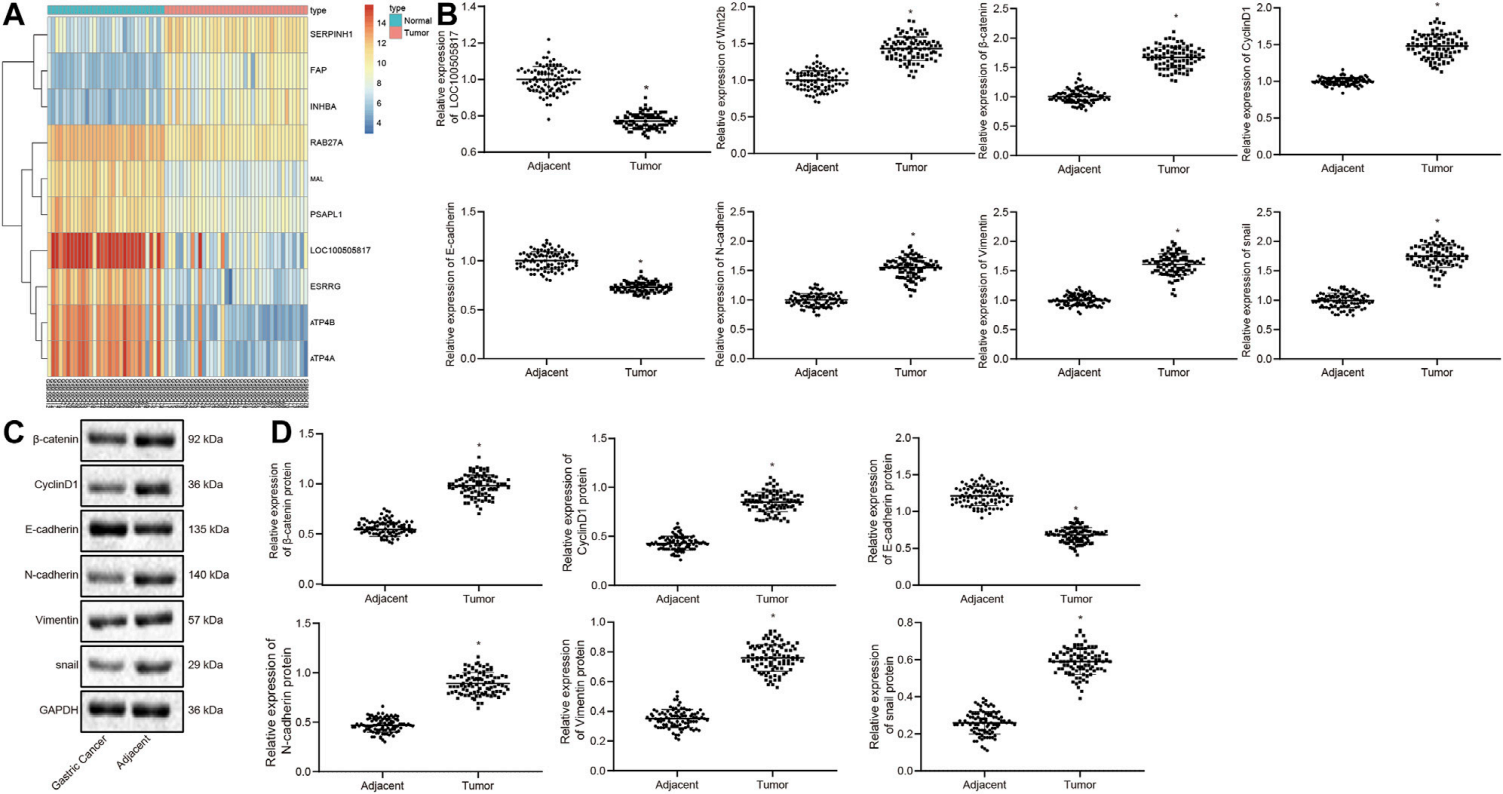
LOC100505817 is downregulated and Wnt/β-catenin signaling pathway is activated in GC tissues. **(A)**, Heat map analysis of the top 10 genes in GSE13911 dataset; **(B)**, LOC100505817 expression and mRNA expression of Wnt2b, β-catenin, CyclinD1, N-cadherin, Vimentin, snail, and E-cadherin in GC tissues and adjacent tissues, as detected by RT-qPCR; *n* = 92; **(C)**, the protein bands of β-catenin, CyclinD1, Vimentin, N-cadherin, snail, and E-cadherin by western blot analysis; **(D)**, Statistical analysis of **(C)**; *n* = 92; ^*^, *p* < 0.05 *vs.* adjacent tissues. The data were presented as mean ± standard deviation. Comparisons between GC tissues and adjacent tissues were analyzed using paired *t* test.

By analyzing the GSE13911 microarray, we found a low expression the LOC100505817 was lowly expressed in GC (Figure 1A). In order to verify the result, RT-qPCR and western blot analysis were conducted for the measurement of expression patterns of Wnt2b, β-catenin, CyclinD1, N-cadherin, Vimentin, snail, LOC100505817 as well as E-cadherin in the harvested GC tumor tissues, to investigate the mechanism by which LOC100505817 and Wnt/β-catenin signaling pathway affects GC. The results of RT-qPCR are shown in Figure 1B, revealing that relative to adjacent tissues, the mRNA expression of Wnt2b, β-catenin, CyclinD1, N-cadherin, Vimentin and snail was remarkably enhanced, while LOC100505817 expression and E-cadherin mRNA expression were reduced in GC tissues (all *p* < 0.05). Besides, western blot analysis (Figures 1C,D), illustrated that in comparison with adjacent tissues, the protein expression of β-catenin, CyclinD1, N-cadherin, Vimentin and snail was up-regulated, while E-cadherin was remarkably diminished in GC tissues (all *p* < 0.05).

Next, with the attempt to determine the involvement of LOC100505817 in the progression of GC, the correlation between the LOC100505817 expression and clinical features of the enrolled patients with GC was assessed. As shown in Supplementary Table S1, LOC100505817 expression was related to tumor diameter, LNM and tumor-node-metastasis (TNM) staging (all *p* < 0.05). A significant decrease of LOC100505817 expression was observed in patients with tumor diameter ≥5, LNM, or TNM at III – IV stage when compared with patients with tumor diameter <5, or without LNM, or TNM at I – II stage (all *p* < 0.05). There was no significant difference between LOC100505817 and age, sex and tumor differentiation. These findings evidently revealed that LOC100505817 could potentially be a factor involved in the progression of GC.

### Downregulated LOC100505817 Activates Wnt/β-Catenin Signaling Pathway to Promote EMT in GC

**FIGURE 2:**
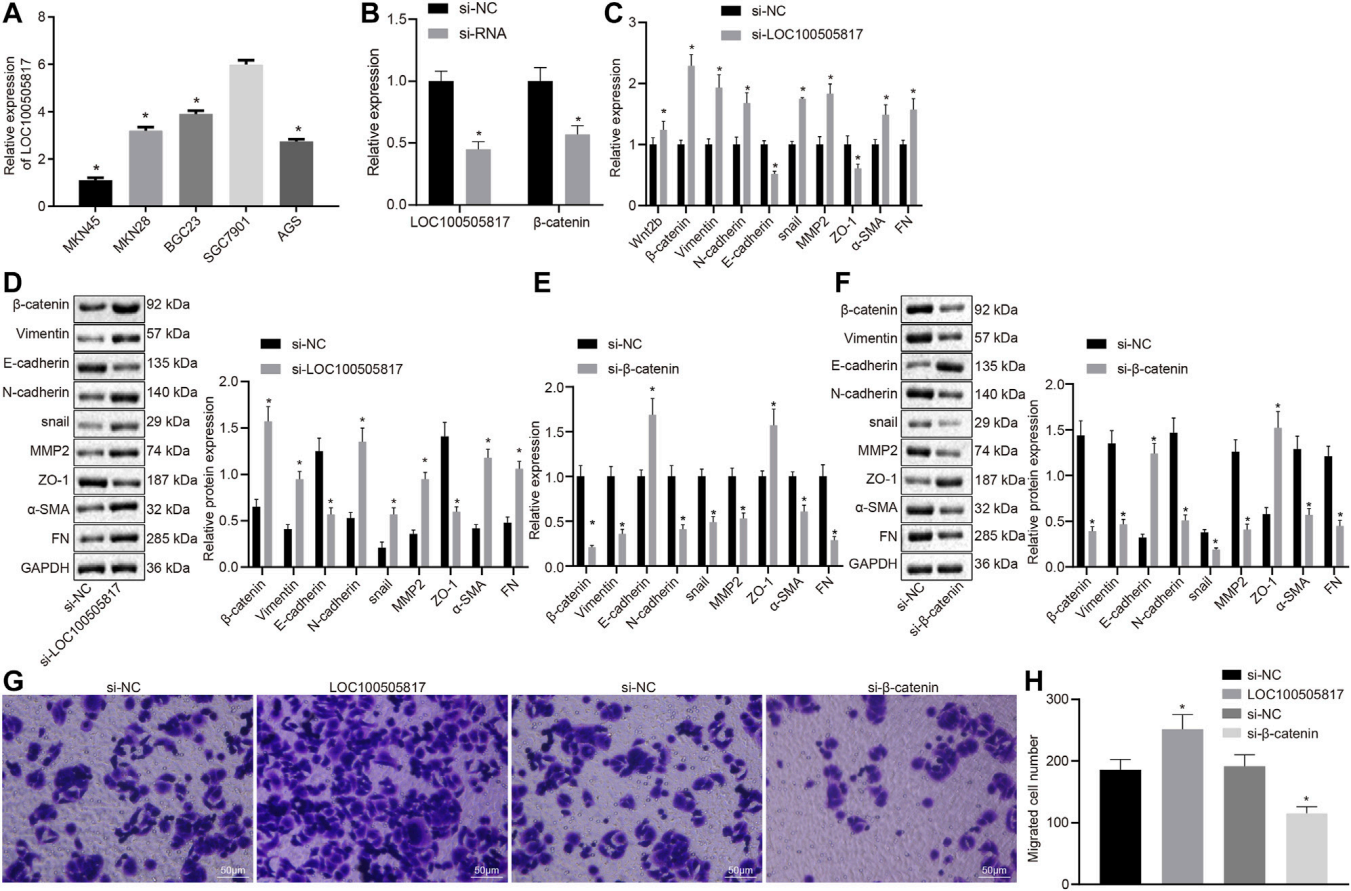
LOC100505817 silencing promotes EMT by activating Wnt/β-catenin signaling pathway in GC SGC7901 cells. **(A)**, LOC100505817 expression in the five GC cell lines by RT-qPCR; **(B)**, LOC100505817 expression and β-catenin mRNA expression in the SGC7901 cells; **(C)**, the mRNA expression of β-catenin and EMT-related markers in SGC7901 cells after silencing LOC100505817 determined by RT-qPCR; **(D)**, the protein expression of β-catenin and EMT-related markers in SGC7901 cells after silencing LOC100505817 determined by western blot analysis; **(E)**, the mRNA expression of β-catenin and EMT-related markers in SGC7901 cells after silencing β-catenin determined by RT-qPCR; **(F)**, the protein expression of β-catenin and EMT-related markers in SGC7901 cells after silencing β-catenin determined by western blot analysis; **(G)**, Representative images of cell invasive ability of SGC7901 cells after silencing β-catenin or LOC100505817 detected by Transwell assay; **(H)**, Histogram of GC cell invasion shown in **(G)**; ^**^, *p* < 0.05 *vs.* the si-NC group. The data were measurement data and presented as mean ± standard deviation. Data between two groups were compared by unpaired *t*-test. The experiments were repeated three times.

LOC100505817 expression was detected in five different GC cell lines (BGC823, AGS, MKN45, SGC7901, and MKN28) by RT-qPCR. The expression of LOC100505817 in BGC823 cells was 3.9 ± 0.14, in AGS cells was 2.75 ± 0.09, in MKN45 cells was 1.1 ± 0.11, in SGC7901 cells was 6.0 ± 0.18 and in MKN28 cells was 3.2 ± 0.15 (Figure 2A). The expression level of LOC100505817 was the highest in SGC7901 cells among five cell lines; therefore, SGC7901 cell line was selected for subsequent experiments.

Next, the correlation of LOC100505817 and Wnt/β-catenin with EMT process was further detected in GC cells. First, LOC100505817 and β-catenin were silenced in SGC7901 cells by siRNA. RT-qPCR results revealed that after siRNA treatment, LOC100505817 and β-catenin expression was decreased in SGC7901 cells (Figure 2B). RT-qPCR and western blot analysis were conducted to detect expression of EMT-related markers in SGC7901 cells. As shown in Figures 2C–F, compared with the si-NC groups, the mRNA (Figures 2C,D) and protein (Figures 2E,F) expressions of β-catenin, N-cadherin, Vimentin, Snail, MMP2, α-SMA and FN in the si-LOC100505817 group were significantly elevated, while that of E-cadherin and ZO-1 was significantly lowered (all *p* < 0.05), and the si-β-catenin group presented with the opposite results.

Then, we further detected the effects of LOC100505817 and Wnt/β-catenin signaling pathway on the migration of GC cells *in vitro* by Transwell assay. The results demonstrated that compared with the si-NC groups, the migration ability of SGC7901 cells in si-LOC100505817 group was significantly increased, while that of SGC7901 cells were significantly reduced in the si-β-catenin group (all *p* < 0.05; Figures 2G,H).

It suggests that LOC100505817 and E-cadherin are down-regulated while Wnt2b, β-catenin, CyclinD1, N-cadherin, Vimentin and snail are potentiated in GC tissues, thus contributing to the EMT.

### Overexpressed LOC100505817 Downregulates β-catenin to Restrain GC SGC7901 Cell Proliferation

**FIGURE 3:**
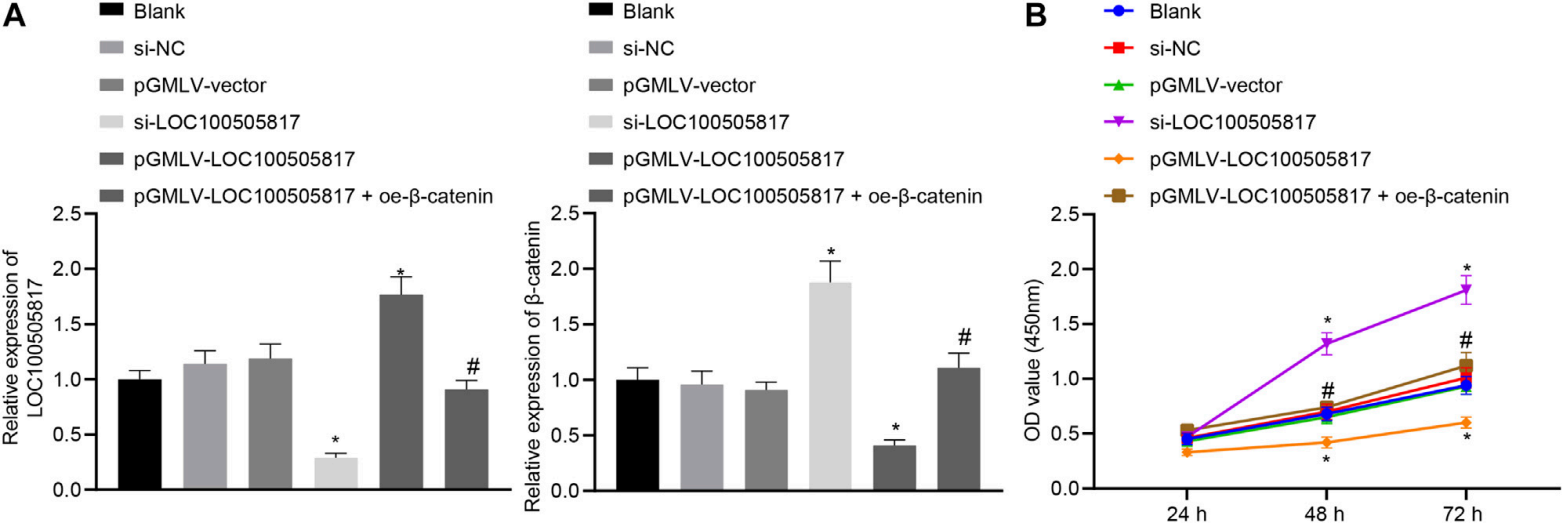
LOC100505817 inhibits GC cell proliferation. **(A)**, LOC100505817 expression in SGC7901 cells after alteration of LOC100505817 detected by RT-qPCR; **(B)**, SGC7901 cell viability after alteration of LOC100505817 detected by CCK-8; ^**^ *p* < 0.05 *vs.* the si-NC group; ^#^ *p* < 0.05 *vs.* the pGMLV-LOC100505817 group. The data were presented as mean ± standard deviation. Comparisons between two groups were analyzed using one way ANOVA, and comparisons among multiple groups at different time points were analyzed by two-way with Bonferroni post-hoc test. The experiment was repeated three times.

Subsequently, we assessed the roles of LOC100505817 on GC cell proliferation. First, the efficiency of lentivirus transfection and β-catenin mRNA expression were detected by RT-qPCR. The results showed that the LOC100505817 expression was evident lower and the mRNA expression of β-catenin was obvious higher in the si-LOC100505817 group than that in the si-NC group, while the LOC100505817 expression was obvious higher and the mRNA expression of β-catenin was significant lower in the pGMLV-LOC100505817 group than that in the pGMLV-vector group (both *p* < 0.05; Figure 3A). Then, as CCK8 assay displayed (Figure 3B), the blank, si-NC and pGMLV-vector groups exhibited no significant difference in the growth rate of SGC7901 cells (all *p >* 0.05). The growth rate of SGC7901 was markedly accelerated in the si-LOC100505817 group, and the OD value at the 48th and 72nd h was significantly higher than that in the blank and si-NC groups (all *p <* 0.05). The growth rate of SGC7901 obviously reduced in the pGMLV-LOC100505817 group, in which the OD value at the 48th and 72nd h was significantly higher than that in the blank and pGMLV-vector groups (all *p <* 0.05). Besides, it was also observed that relative to the pGMLV-LOC100505817 group, the inhibited cell proliferation was reversed in the pGMLV-LOC100505817 + oe-β-catenin group (all *p* < 0.05). These data suggest that LOC100505817 has an inhibitory effect on GC SGC7901 cell proliferation through downregulating β-catenin.

### Overexpressed LOC100505817 Inhibits GC SGC7901 Cell Migration and Invasion

**FIGURE 4:**
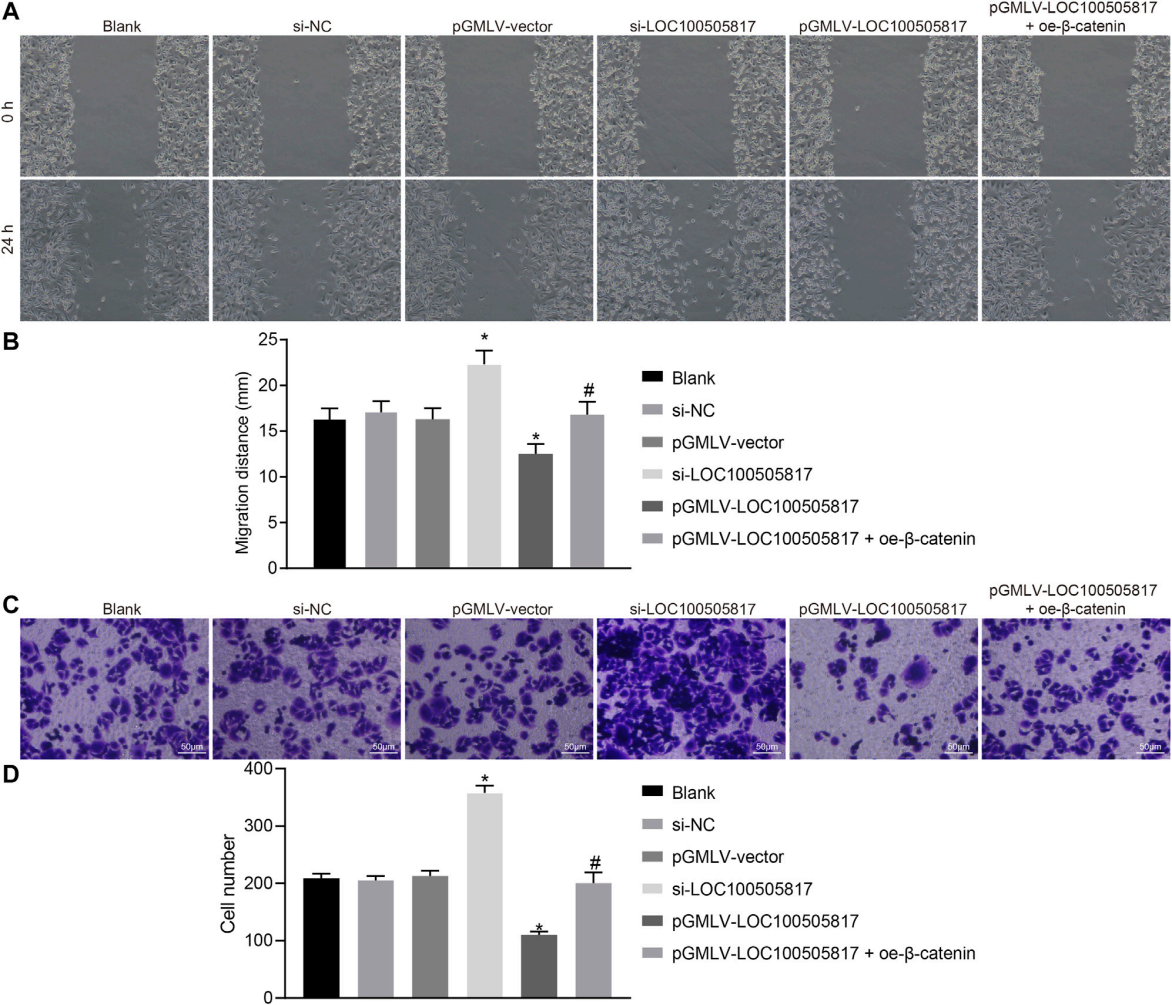
LOC100505817 inhibits GC cell migration and invasion. **(A)**, Representative images of cell migratory ability detected by wound healing assay; **(B)**, Histogram of GC cell migration distance shown in **(A)**; **(C)**, Representative images of cell invasive ability detected by Transwell assay; **(D)**, Histogram of GC cell invasion shown in **(C)**; ^**^ *p* < 0.05 *vs.* the si-NC group; ^##^ *p* < 0.05 *vs.* the pGMLV-LOC100505817 group. The data were presented as mean ± standard deviation, which were analyzed using one way ANOVA; the experiment was repeated three times.

Next, in order to determine GC cell migration and invasion influenced by LOC100505817, starch test and Transwell assay were used. Results of scratch test (Figures 4A,B) showed that after 24 h, no significant difference was observed in cell migration among the blank, si-NC and pGMLV-vector groups (*p >* 0.05). Cell migration in the si-LOC100505817 group was notably promoted, but significantly weakened in the pGMLV-LOC100505817 group versus that in the blank group (both *p* < 0.05). Moreover, as compared with the pGMLV-LOC100505817 group, the inhibited cell migratory capability was reversed in the pGMLV-LOC100505817 + oe-β-catenin group (all *p* < 0.05). It indicates that GC SGC7901 cell migration is inhibited by LOC100505817 overexpression.

The results of Transwell assay revealed no evident difference in cell invasion among the blank, si-NC and pGMLV-vector groups (*p >* 0.05). Cell invasion in the si-LOC100505817 group was remarkably enhanced, but significantly repressed in the pGMLV-LOC100505817 group relative to that in the blank group (all *p* < 0.05). In addition, the inhibited cell invasive capability was reversed in the pGMLV-LOC100505817 + oe-β-catenin group than in the pGMLV-LOC100505817 group (all *p* < 0.05) (Figures 4C,D). These results led to the conclusion that overexpression of LOC100505817 might suppress GC SGC7901 cell invasion.

### Overexpressed LOC100505817 Blocks GC SGC7901 Cell Cycle Entry and Induces GC SGC7901 Cell Apoptosis

**FIGURE 5:**
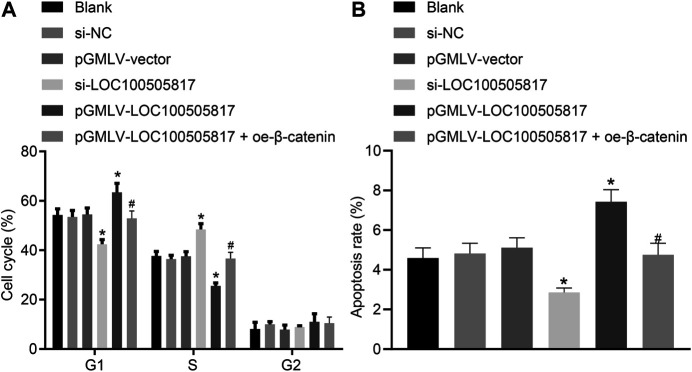
LOC100505817 accelerates GC cell apoptosis and arrests GC cell at G1 phase. **(A)**, Cell cycle distribution in the cells after transfection; **(B)**, Cell apoptosis of the cells after transfection; GC cells in the scatter plots in which the upper left quadrant identifies the necrotic cells (annexin V−/PI+), the upper right quadrant identifies the late apoptotic cells (annexin V+/PI+), the lower left quadrant identifies the live cells (annexin V−/PI−), and the lower right quadrant identifies the early apoptotic cells (annexin V+/PI−); ^*^ *p* < 0.05 *vs.* the si-NC group; ^#^ *p* < 0.05 *vs.* the pGMLV-LOC100505817 group. The data were presented as mean ± standard deviation, which were analyzed using one way ANOVA. The experiment was repeated three times.

Furthermore, for the purpose to detect the functions of LOC100505817 on GC cell apoptosis and cell cycle, flow cytometry was employed. PI single staining results (Figure 5A) showed that, in the blank, si-NC, pGMLV-vector, si-LOC100505817, and pGMLV-LOC100505817 groups, the cell proportions at G1 phase were (54.26 ± 2.52), (53.53 ± 2.54), (54.52 ± 2.57), (42.53 ± 1.77), and (63.43 ± 3.69)%, respectively; cell proportions at S phase were (37.65 ± 1.86), (36.43 ± 1.54), (37.57 ± 1.89), (48.53 ± 2.23), and (25.54 ± 1.25)%, respectively; cell proportions at G2 phase were (8.09 ± 4.38), (10.04 ± 4.08), (7.91 ± 4.46), (8.94 ± 4.00), and (11.03 ± 4.94)% in proper order. The blank, si-NC and pGMLV-vector groups illustrated no significant difference in cell cycle distribution (*p >* 0.05). In comparison with the blank and si-NC groups, cell number in G1 phase was decreased in the si-LOC100505817 group yet increased in S phase (all *p* < 0.05). The pGMLV-LOC100505817 group was observed to have increased number of cells in the G1 phase and decreased cells in the S phase (all *p* < 0.05). Compared with the pGMLV-LOC100505817 group, the pGMLV-LOC100505817 + oe-β-catenin group showed decreased cell number in G1 phase and increased cell number in S phase (all *p* < 0.05).

The results obtained from Annexin V/PI double staining (Figure 5B) confirmed that apoptosis rates in the blank, si-NC, pGMLV-vector, si-LOC100505817, and pGMLV-LOC100505817 groups at 48 h after transfection were (4.59 ± 0.51), (4.82 ± 0.52), (5.11 ± 0.50), (2.86 ± 0.22) and (7.43 ± 0.61)%, respectively. Consistently, no significant difference was observed in apoptosis rate among the blank, si-NC and pGMLV-vector groups (*p* > 0.05). Apoptosis rate was reduced significantly in the si-LOC100505817 group while notably enhanced in the pGMLV-LOC100505817 group versus that in the blank and si-NC groups (all *p* < 0.05). Moreover, relative to the pGMLV-LOC100505817 group, the cell apoptosis was observed to decrease in the pGMLV-LOC100505817 + oe-β-catenin group (all *p* < 0.05). These results suggest that overexpressed LOC100505817 can accelerate GC SGC7901 cell apoptosis and arrest GC SGC7901 cell at G1 phase.

### LOC100505817 Represses Wnt/β-Catenin Signaling Pathway Activation and EMT Progression in GC SGC7901 Cells

**FIGURE 6:**
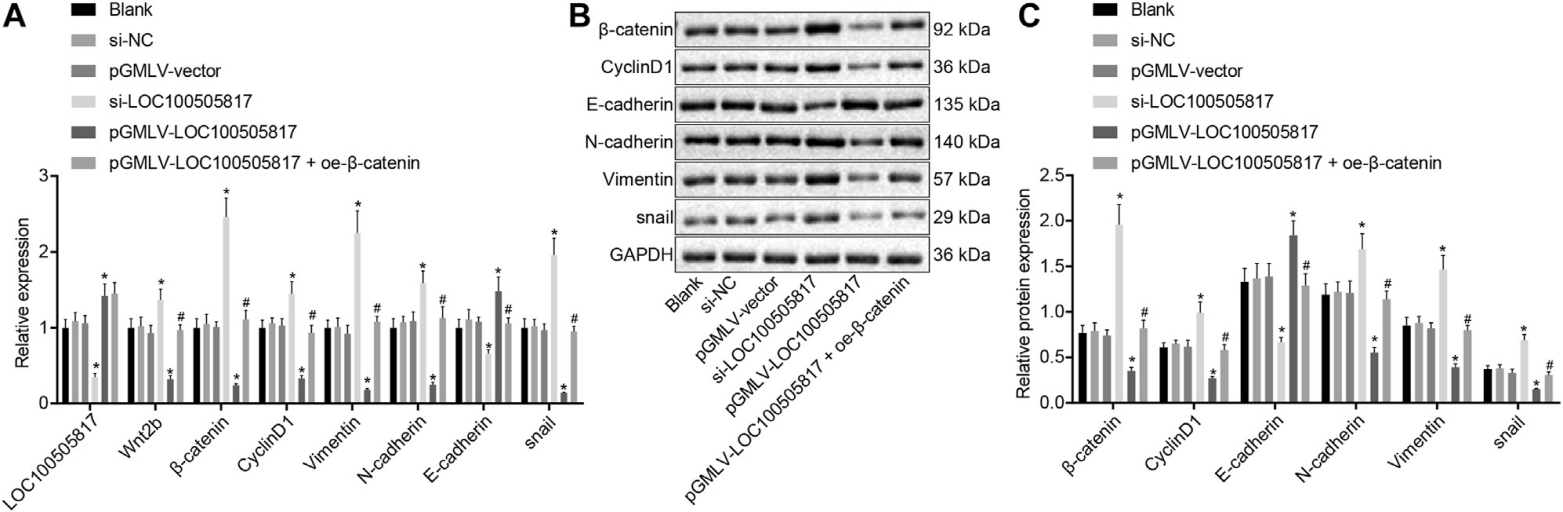
Overexpressed LOC100505817 down-regulates the Wnt/β-catenin signaling pathway and EMT in GC cells. **(A)**, RT-qPCR detection on the LOC100505817 expression and mRNA levels of Wnt2b, β-catenin, CyclinD1, Vimentin, N-cadherin, snail, and E-cadherin; **(B)**, the protein bands of β-catenin, CyclinD1, Vimentin, N-cadherin, snail, and E-cadherin by western blot analysis; **(C)**, Statistical analysis of **(B)**; ^**^ *p* < 0.05 *vs.* the si-NC group; ^##^ *p* < 0.05 *vs.* the pGMLV-LOC100505817 group. The data were presented as mean ± standard deviation, which were analyzed using one way ANOVA. The experiment was repeated three times.

Finally, with the attempt to explore the impacts of LOC100505817 on Wnt/β-catenin signaling pathway and EMT, RT-qPCR and western blot analysis were performed to detect expression of LOC100505817, Wnt2b, β-catenin, CyclinD1, Vimentin, N-cadherin, E-cadherin as well as snail. As displayed in Figure 6, there was no significant difference in LOC100505817 expression and mRNA and protein expression of Wnt2b, β-catenin, CyclinD1, Vimentin, N-cadherin, E-cadherin and snail were shown among the blank, si-NC and pGMLV-vector groups (all *p* > 0.05). In comparison with the blank group, expression of Wnt2b, β-catenin, CyclinD1, Vimentin, N-cadherin and snail was remarkably up-regulated while that of LOC100505817 and E-cadherin was notably diminished in the si-LOC100505817 group (all *p* < 0.05). Contrary to that, a profound decline was shown in the expression of Wnt2b, β-catenin, CyclinD1, Vimentin, N-cadherin and snail in the pGMLV-LOC100505817 group, with a dramatical enhancement in expression of LOC100505817 and E-cadherin (all *p* < 0.05). When compared with the pGMLV-LOC100505817 group, the expression of Wnt2b, β-catenin, CyclinD1, Vimentin, N-cadherin and snail was elevated in the pGMLV-LOC100505817 + oe-β-catenin group, while expression of E-cadherin was significantly reduced (all *p* < 0.05). Therefore, LOC100505817 might potentially contribute to the suppression of the Wnt/β-catenin signaling pathway and EMT.

## Discussion

Gastric cancer has a high incidence and mortality worldwide [27, 28]. Accumulating evidence has highlighted the role of lncRNAs as crucial regulators in tumorigenesis and development, which can be applied as a novel method in the inhibition of tumorigenesis and invasion of GC [5, 29]. The current study was conducted with emphasis placed on the relationship between LOC100505817 expression and GC. This was done by analyzing differential expression of LOC100505817, the results of which indicated the inhibitory role of LOC100505817 in GC and provided a preliminary theoretical basis to aid the improvement of current diagnostic methods and delay tumor development of GC.

We initially demonstrated that elevation of LOC100505817 expression resulted in the significant suppression of GC progression through the inhibition of the proliferation, migration and invasion while inducing apoptosis of GC SGC7901 cells. LncRNA are recognized as novel regulators in the cancer paradigm and may possess potential values in oncogenic as well as tumor suppressive pathways and a wide range of human tumors have been found to have dysregulated lncRNA [30]. A prior study revealed the presence of reduced levels of LINC00675 in GC, playing a tumor suppressor role through the collapse of vimentin filaments [31]. GC tissues have poor expressions of numerous lncRNAs, such as lncRNA uc001lsz, HMlincRNA717, lncRNA BM709340, BQ213083, AK054978, DB077272, lncRNA-ATB and lncRNA-ZEB1 [29, 32]. Furthermore, GSE13911 data revealed downregulated levels of LOC100505817 in GC. In addition, a mount number of lncRNAs including lncRNA-ANRIL, lncRNA MEG3, lncRNA-MIAT and lncRNA-AK058003 have been identified as key regulators of several biological behaviors, including proliferation, apoptosis, invasion as well as metastasis of GC [33–37]. The specific mechanism by which LINC00675 impedes the biological process of proliferation, migration and invasion, and accelerates apoptosis of GC involves the enhancement of p53 signaling pathway activity [31]. Similarly, the knockdown of LINC00152 and lncRNA HOTAIR in GC cells has been demonstrated to promote cell cycle and trigger late apoptosis [38, 39]. Moreover, our findings also showed that overexpressed LOC100505817 led to the inhibition of GC cell proliferation, migration and invasion, while enhancing cell apoptosis.

Our data also indicated that the expression of relevant genes linking to Wnt/β-catenin signaling pathway and EMT was elevated secondary to LOC100505817 silencing. A previous study also suggested that the Wnt-1, Wnt-2 and Wnt-2b, proteins in the Wnt family, are up-regulated in GC, and the up-regulation of Wnt-2 was positively correlated to an increase in metastatic potential [40]. Wnt/β-catenin signaling has also been linked with the telomerase subunit Tert and naturally implicated in cancer, due to the fundamental association of telomerase activity to cancer development [41]. Activated Wnt/β-catenin signaling pathway is implicated in the progression of various human cancers, including GC [42]. Moreover, the Wnt/β-catenin signaling pathway is responsible for regulating numerous lncRNAs, including LncRNA PTCSC3, lncRNA SNHG20 and lncRNA TUG1 [43–45]. Furthermore, LOC100505817 was confirmed to be capable of suppressing the activation of the Wnt/β-catenin signaling pathway in the present study. It was reported that the Wnt/β-catenin signaling pathway participates in the cellular process of GC and disturbances of Wnt/β-catenin signaling pathway can suppress the growth, invasion in addition to metastasis of GC [40, 46]. In breast cancer, Wnt3 overexpression can promote EMT-like phenotype through activating the Wnt/β-catenin signaling pathway [47]. EMT involves the down-regulation of E-cadherin and plays a vital role during the early stage of cancer metastasis [32, 34, 48]. EMT may result in the loss of intercellular connections of cancer cells, upregulation of contractile cytoskeleton components and remove the benign primary on account of epithelium; these changes make EMT a key factor that should not be neglected when studying the development of cancer [49]. LncRNAs can regulate EMT and the silencing of lncRNA HOTAIR could exert an inhibitory role on EMT in GC [39, 49]. In addition, our study revealed that LOC100505817 inhibited EMT by lowering Vimentin, N-cadherin and snail and restoring E-cadherin expression.

## Conclusion

Collectively, the findings obtained in this study summarized that human GC cells have a low expression of LOC100505817. Ectopic expression of LOC100505817 correlated with cellular process of GC. These results further highlight the need to focus on LOC100505817 as a potential biomarker for GC. However, additional studies are recommended in order to lay a theoretical foundation for better prevention, diagnosis, and treatment measures on a genetic level.

## Data Availability

The original contributions presented in the study are included in the article/Supplementary Material, further inquiries can be directed to the corresponding author.
