# Supplementary material for: Tumor Inhibitory Effect of Long Non-coding RNA LOC100505817 on Gastric Cancer
Source: Pathol Oncol Res. 2021 May 26;27:581542. doi: 10.3389/pore.2021.581542 (PMC8354317; doi:10.3389/pore.2021.581542)
Supplement: Supplementary file 1 [file Table1.docx]

**Supplementary Table 1** Correlation between the expression of LOC100505817 and clinical characteristics of GC

| Clinical-pathological parameters | Cases | LOC100505817 expression | *p* value |
| --- | --- | --- | --- |
|  |  | Mean value |  |
| Age (years) |  |  | 0.235 |
| ≥ 60 | 49 | 0.32 ± 0.04 |  |
| < 60 | 43 | 0.33 ± 0.04 |  |
| Sex |  |  | 0.234 |
| Male | 48 | 0.32 ± 0.04 |  |
| Female | 44 | 0.32 ± 0.04 |  |
| Tumor diameter (cm) |  |  | 0.018 |
| < 5 | 38 | 0.33 ± 0.05 |  |
| ≥ 5 | 54 | 0.31 ± 0.03 |  |
| LNM |  |  | 0.010 |
| Yes | 55 | 0.31 ± 0.04 |  |
| No | 37 | 0.33 ± 0.04 |  |
| TNM staging |  |  | 0.001 |
| I - II | 32 | 0.34 ± 0.04 |  |
| III - IV | 60 | 0.31 ± 0.04 |  |
| Degree of tumor differentiation |  |  | 0.236 |
| High-differentiation or moderate differentiation | 50 | 0.32 ± 0.04 |  |
| Poor differentiation or no differentiation | 42 | 0.33 ± 0.04 |  |

Note: TNM, tumor-node-metastasis; LNM, lymph node metastasis; comparisons of the data were analyzed with independent sample *t*-test**.**
